# Triple Threat: Triple-Valve Endocarditis Case Report and Literature Review

**DOI:** 10.14797/mdcvj.1277

**Published:** 2023-10-12

**Authors:** Kiriti S. Vattikonda, Christopher J. Peterson, Jordan L. Torres, Michael Sternberg, Ijeoma Okogbue, Anthony Baffoe-Bonnie, Tasaduq Fazili

**Affiliations:** 1 Virginia Tech School of Medicine, Roanoke, Virginia, US

**Keywords:** endocarditis, triple valve, multiple valve, MSSA

## Abstract

Triple valve endocarditis (TVE) is a rare presentation of endocarditis often requiring multivalvular surgery. Here we report a case of S. *aureus* triple valve endocarditis in a patient with a history of intravenous drug use and provide a literature review of TVE identification, treatment, and prognosis.

## Introduction

Infective endocarditis (IE) causes significant inflammation of the endocardium that can lead to serious cardiac and infectious pathology. Despite major advancements in diagnostic procedures and therapeutic interventions, IE is still associated with significant morbidity and mortality. One retrospective cohort study noted a high in-hospital mortality rate of 18% and a 6-month mortality of 27%.^1^ Risk factors include intravenous (IV) drug use, preexisting valvular disease, hemodialysis, presence of intracardiac devices, diabetes mellitus, and immunosuppression. The majority of IE cases involve a single valve with double-valve involvement being less frequent. Triple (TVE) or quadruple valve endocarditis is rare and infrequently described in the literature. Here we describe a case of TVE in an IV drug user^2^ and provide a literature review of TVE identification, treatment, and prognosis.

## Case Report

**Figure 1:**
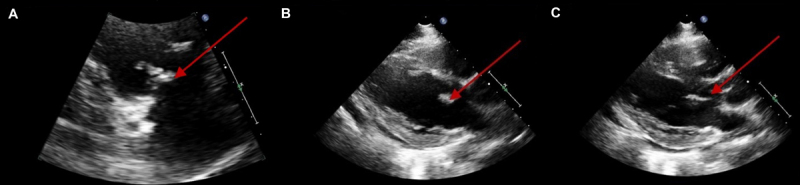
Transthoracic echocardiography of trivalvular vegetation. Vegetations observed on transesophageal echocardiography were as follows: **(A)** tricuspid valve (16 × 8.1 mm), **(B)** mitral valve (13.6 × 8.0 mm), and **(C)** aortic valve (18.3 × 4.7 mm).

A 37-year-old man was evaluated for behavior changes after being tossed from an unknown vehicle onto a driveway 4 days earlier. He had completed treatment for methicillin-resistant *Staphylococcus aureus* infective endocarditis 2 months prior but continued to use IV heroin. He was intubated for airway protection and started on broad-spectrum antibiotics with vancomycin and cefepime. A transthoracic echocardiogram (TTE) identified vegetations on the aortic, mitral, and tricuspid valves (Figure 1) and revealed moderate tricuspid valve regurgitation and mild aortic valve regurgitation.

**Figure 2:**
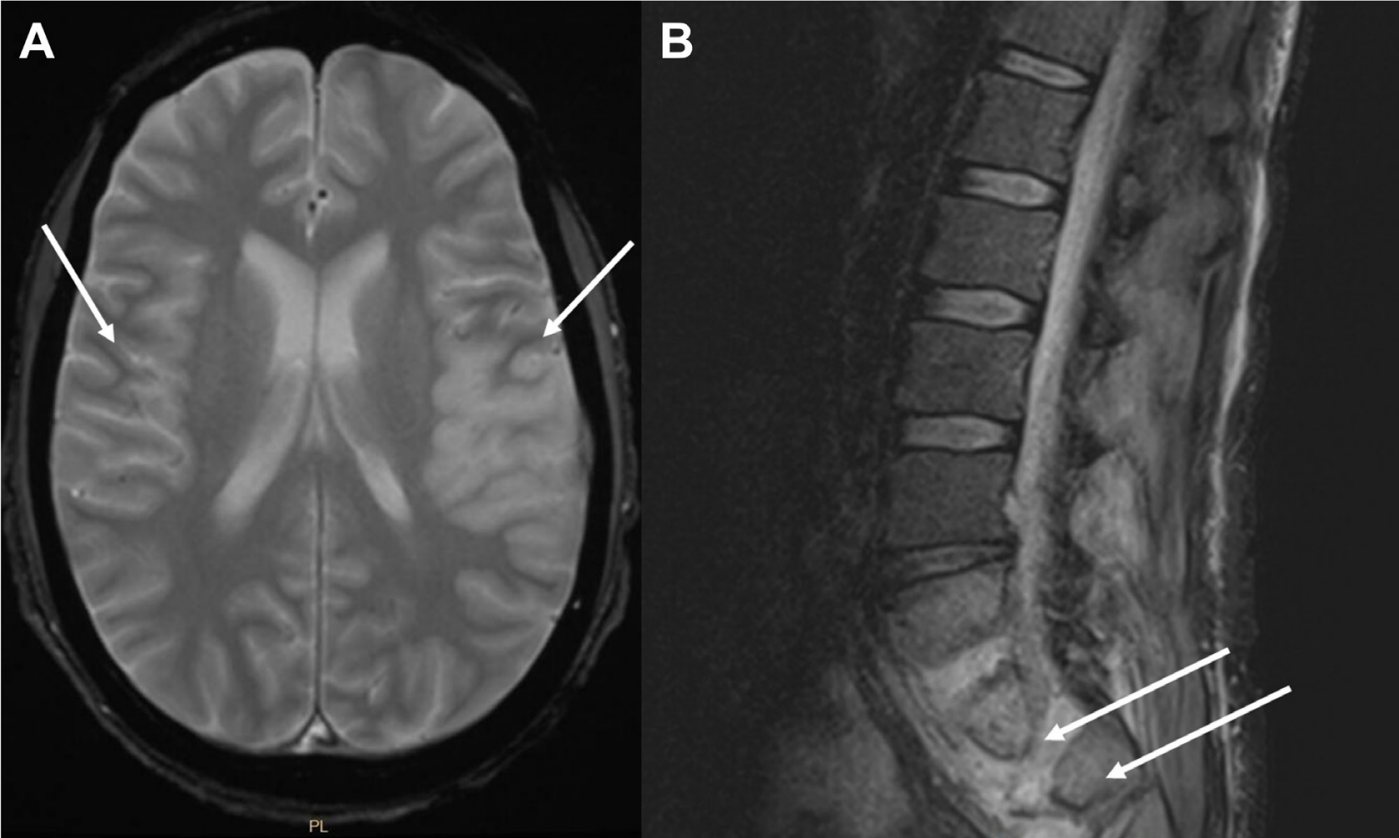
Brain and spinal cord infarctions and abscess. **(A)** Magnetic resonance (MRI) of brain with and without (w/wo) contrast with left and right middle cerebral artery infracts and **(B)** L spine MRI showing L5-S1 discitis-osteomyelitis with intramuscular paraspinal abscess at the L5 and S1 levels.

Magnetic resonance imaging (MRI) of the lumbar spine revealed evidence of vertebral osteomyelitis and extensive intramuscular paraspinal abscesses at the L5 – S1 levels (Figure 2). MRI of the brain showed multiple areas of acute infarction involving the left anterior cerebral, left middle cerebral, and right middle cerebral arterial territories (Figure 2). Given involvement of bilateral vascular territories, a cardiac embolic source was thought to be the likely contributing etiology. Blood cultures obtained on arrival grew methicillin-sensitive *S. aureus* and the patient’s antibiotic regimen was transitioned to IV nafcillin. His hospital course was complicated by the development of bilateral pulmonary emboli, acute renal failure, and profound thrombocytopenia. Cardiothoracic and neurosurgery teams evaluated the patient and deemed him a poor surgical candidate; as such, a transesophageal echo (TEE) was deferred. Due to extensive embolic disease, multiple metastatic sites of infection, and continued lack of neurologic response, his family had the patient terminally extubated on day 3 of hospitalization.

## Discussion

Triple valve endocarditis represents an infrequent but challenging presentation of endocarditis. Given its rarity, there are no randomized control trials or societal guidelines on its management. Most of our understanding of TVE comes from limited clinical case reports, with a 2018 paper noting only 16 published cases.^2^ Given this, much of the data regarding epidemiology and management of TVE is derived from a broader category of multivalvular endocarditis (MVE), which incorporates infections involving two or more valves. MVE is also infrequent, though rates of up to 18% have been identified among endocarditis cases.^3^ Data regarding both TVE and the broader category MVE are used here to help elucidate this rare pathology. Diagnosis of TVE is typically made by echocardiography; however, TTE only detects 50% of cases and TEE is often required for confirmation.^4^ The majority of TVE cases involve the aortic, tricuspid, and mitral valves.^2^ Surgical intervention is commonly needed for TVE patients. A study of 680 endocarditis patients, including 115 MVE, did not find statistically significant differences in patient demographics, predisposing conditions, or previous heart disease.^5^ Regarding pathogens, *Staphylococcus aureus* and *Streptococcus* species are unsurprisingly the most common organisms isolated in MVE and occur at similar rates compared to single-valve endocarditis.^5^ However, unique cases of infections with organisms such as *Mycobacterium tuberculosis* have also been reported.^6^

Surgical intervention plays an important role in TVE management and arguably to a greater degree than in single-valve endocarditis, likely due to more extensive cardiac pathology. A review of 15 cases by Kabado et al. notes that 90% of TVE patients had valve replacement surgery, most of which involved multiple valves.^2^ A study of 680 endocarditis patients discussed by Lopez et al. found that MVE was more frequently associated with surgery (70% of MVE cases vs 54% of SVE cases, *P* = .002).^5^ In another study described by Mihaljevic et al. looking at 63 patients with MVE, it was noted that surgical treatment was associated with favorable early and late mortality rates and satisfactory postoperative functional status, and early intervention before abscess formation improved mortality.^7^

It is important to note, however, that the number of valves involved is not an established indication for surgery. Furthermore, mortality rates can be high, with one center observing a multivalve operative mortality rate of 16% over a 24-year period.^7^ On the other hand, a study of 90 endocarditis patients with surgical valve repair found no difference in complications between those receiving single and multiple valve repairs.^8^ Nevertheless, in cases such as those described here, patients may be too ill to receive valvular surgery. Preoperative renal failure has been found to be a predictor of hospital mortality in MVE surgical patients^4^ and could be used to risk-stratify patients. Patients have improved with medical therapy alone.^9,10^

It is unclear to what extent MVE results in worse outcomes compared with SVE. For example, two studies found that only congestive heart failure (CHF) was significantly increased compared to SVE (65% vs 50%, *P* = .003; 16% vs 64%, *P* = .002).^5^ However, a study of 79 patients found a statistically significant increase in mortality with increasing valve involvement (two valves: HR 4.73, *P* = .006; three valves: HR 14.19, *P* = .014).^3,11^ Heart failure has been shown as an independent predictor of mortality in MVE patients.^5^ Heart failure, among all other indications of surgical intervention, is also the most crucial in prompting intervention. In the presence of CHF, mortality in native MVE is very high (55-85%) with medical management and much lower (10-35%) with surgery.^2^ It may be worth noting that the high incidence of CHF occurring in MVE may lead to higher rates of surgical intervention, resulting in lower-than-expected mortality rates in complex disease.^12^ Ultimately, endocarditis involving multiple valves, such as a triple valve presentation as described here, are likely to be complex and require careful consideration of surgical and medical treatment options.

## Conclusion

TVE is a rare though serious manifestation of infectious endocarditis. Cases often require surgical management, including multivalve replacement, although some patients may be too critically ill for surgical intervention. As with any form of endocarditis, physicians should have a high index of suspicion for embolic phenomena. Finally, although no official guidelines for surgical management of TVE exist, early surgical consultation may be prudent given the frequency of valve replacement for these patients.
